# Assessment of Genetic Diversity of Sweet Potato in Puerto Rico

**DOI:** 10.1371/journal.pone.0116184

**Published:** 2014-12-31

**Authors:** Lorraine Rodriguez-Bonilla, Hugo E. Cuevas, Milly Montero-Rojas, Fernando Bird-Pico, Dianiris Luciano-Rosario, Dimuth Siritunga

**Affiliations:** 1 Department of Biology, University of Puerto Rico Mayaguez, Mayaguez, Puerto Rico, United States of America; 2 Tropical Agriculture Research Station, US Department of Agriculture–Agricultural Research Service, Mayaguez, Puerto Rico, United States of America; The University of Western Australia, Australia

## Abstract

Sweet potato (*Ipomoea batatas* L.) is the seventh most important food crop due to its distinct advantages, such as adaptability to different environmental conditions and high nutritional value. Assessing the genetic diversity of this important crop is necessary due to the constant increase of demand for food and the need for conservation of agricultural and genetic resources. In Puerto Rico (PR), the genetic diversity of sweet potato has been poorly understood, although it has been part of the diet since Pre-Columbus time. Thus, 137 landraces from different localities around PR were collected and subjected to a genetic diversity analysis using 23 SSR-markers. In addition, 8 accessions from a collection grown in Gurabo, PR at the Agricultural Experimental Station (GAES), 10 US commercial cultivars and 12 Puerto Rican accessions from the USDA repository collection were included in this assessment. The results of the analysis of the 23 loci showed 255 alleles in the 167 samples. Observed heterozygosity was high across populations (0.71) while measurements of total heterozygosity revealed a large genetic diversity throughout the population and within populations. UPGMA clustering method revealed two main clusters. Cluster 1 contained 12 PR accessions from the USDA repository collection, while cluster 2 consisted of PR landraces, US commercial cultivars and the PR accessions from GAES. Population structure analysis grouped PR landraces in five groups including four US commercial cultivars. Our study shows the presence of a high level of genetic diversity of sweet potato across PR which can be related to the genetic makeup of sweet potato, human intervention and out-crossing nature of the plant. The history of domestication and dispersal of sweet potato in the Caribbean and the high levels of genetic diversity found through this study makes sweet potato an invaluable resource that needs to be protected and further studied.

## Introduction

The conservation of crop genetic resources is vital for food security. The on-going abuse of urbanization and construction is responsible for the everyday loss of valuable land for agricultural production with wild-relatives of important crops being lost forever due to deforestation. These wild-relatives could have important traits useful for crop improvement, bio-fortification programs or the conservation of genetic resources. Maintaining a healthy genetic pool and preventing genetic erosion can positively impact the economy and overall food production. There is immense uncertainty as to whether the increase in world food production could be met without studying and protecting this diversity [1].

Sweet potato (*Ipomoea batatas* (L.) Lam.) is the seventh most important food crop after wheat, rice, maize, potato, barley and cassava [2]. This crop has recently received greater research-related attention due to its many agricultural advantages such as its adaptability to different environmental conditions and its nutritional value, being an excellent source of carbohydrates, dietary fiber, sugars, proteins, iron and calcium. In addition, it is also an important source of vitamin A and C, especially in the orange-fleshed varieties making sweet potato a key crop to solve the vitamin A deficiencies around the world. Vitamin A deficiencies lead to the death of more than 600,000 people per year, especially of pregnant women and children in developing countries in Sub-Saharan Africa and South East Asia [3]. With the abundance of undernourishment throughout the world, nutritious crops such as sweet potato needs to be further studied and exploited.

Linguistic and archeological findings indicate that sweet potato was domesticated in America between the mouth of the Orinoco River in Venezuela and the Yucatan peninsula more than 10,000 years ago [4], [5]. Sweet potato was then distributed throughout the Americas by New World inhabitants through migration routes and introduced into Europe after the 1^st^ trip of Christopher Columbus to Americas in 1492 via Spain [4], [6]. On this first trip to America, Columbus visited different islands such as Cuba and Hispaniola (Dominican Republic and Haiti) from where many different plants were acquired in exchange of other resources such as gold. The inhabitants of these islands, Taínos, were descendants of the Arawakan-speaking people from northeastern Venezuela and the French Guiana coast who colonized the West Indies [7]. Interestingly, according to O′Brien [4], the name still used in Puerto Rico and other Caribbean island to describe sweet potato, “batata”, originates from the Arawak language.

In the early part of this century, due to genetic differences of sweet potato in those areas, it was believed that sweet potato was domesticated in Meso-America and then spread to the Peruvian-Ecuadorian region [8], [9]. This extension to other areas suggested the presence of two different gene pools (South America and Central America/Caribbean) supporting the previous theories about the area of sweet potato domestication [8], [9]. However, subsequently new theories have arisen regarding the origin of the domesticated form of sweet potato we know today. The main theory suggests that two independent domestication events occurred: one in Meso-America and the other the North-Western part of South America [10]. The possibility of having two independent domestication events exists by the fact that other crops such as individuals from the genus Cucurbita were domesticated individually in different areas of America, likewise, some wild relatives of *Phaseolus vulgaris* were domesticated twice in different geographical locations [10], [11]. When 459 sweet potato accessions from different localities in America were assessed for their diversity using seven chloroplastic and thirteen nuclear Simple Sequence Repeat (SSR) markers a pattern of division was observed [9], [10]. The samples segregated into two groups, North and South, with North being comprised of samples from Central America and the Caribbean and South comprised of samples from South America. These results give validity to the previously alluded theory that sweet potato could have been domesticated twice, once in the Southern parts of South America and the other in the Northern part of South America/Central America/Caribbean region. The different migration routes and high levels of genetic diversity identified in previous work leads us to believe that the Caribbean is a hot spot for genetic diversity of sweet potato and a potential secondary center for the diversity of this crop.

Historically farmers have relied only on morphological traits in order to distinguish between different cultivars of a crop. In sweet potato, cultivation constraints such as lack of space between cultivars in the field, vine spreading and morphological variation can cause further confusion regarding the exact identity of the cultivars being grown in a field. Such confusions can lead to the disappearance of important genes from the population and loss of genetic diversity. One of the best practices to avoid confusion among cultivars and to correctly catalogue the existing germplasm is the use of molecular markers to understand the genetic composition of any crop. Molecular markers have proven to be very informative and can successfully detect clones or duplicate accessions which were treated as different cultivars. For such studies SSR markers have become the marker of choice due to their highly polymorphic and co-dominant nature, reproducibility and their equal distribution throughout a plant genome [12]. Herein, we assess the genetic diversity of sweet potato in Puerto Rico through the analysis of 23 SSR markers. Understand this genetic diversity is imperative to the establishment of conservation efforts and to warrant the adequate use of the germplasm.

## Materials and Methods

### Plant Material

Sweet potato leaf samples were collected from subsistence farmers and land-owners voluntarily. Root samples were not collected. Any access leaf samples left-over after DNA extraction was destroyed. Since this study did not involve any endangered or protected species specific governmental permission was not required to collect the leaf samples. The sweet potato plant material was obtained from 3 different sources. First, a group comprising 8 known Puerto Rican accessions (Gonzalez, Martina, Camuy, Manolo, Gem, Craneal, Carlos Hernandez and Pujols) was collected from the Agricultural Experimental Station in Gurabo, PR (GAES). Second, twenty two samples comprised of 10 known US commercial cultivars (GemGA, Jewel, Blanquita, Beauregard, Hernandez, Vardaman, Centennial, Porto Rico, Nugget and Bunch Porto Rico) and 12 known Puerto Rican accessions (Miguela-Arecibo, Tapato, Pepa de Oro, Amanecer, Francia, Mojave, Macana, Sunny, Frita, Wart, Buggsbunny and Papota) were obtained from *in-vitro* plants provided by The Plant Genetic Resources Conservation Unit (PGRCU) in Griffin, GA [13]. The last group consisted of 137 samples from across Puerto Rico collected from subsistence farmers or land-owners. The leaf samples of these PR landraces were collected in addition to their geographical location (municipality, community), color and the local name of the sample (if known) (S1 Table). Leaf materials from both old and young leaves were stored at −20°C until DNA was extracted.

### DNA Extraction

DNA extractions were performed using leaf tissue according to the method described by Doyle and Doyle (1990) with some modifications [14]. Approximately 30 mg of leaf tissue was ground using a sterile pestle and sea sand in a 2.0 mL tube for 3 minutes followed by the addition of 700 µL of 2% CTAB extraction Buffer [20 mM EDTA, 0.1 M Tris-HCl pH 8.0, 1.4 M NaCl, 2% CTAB] and further grounding for 2 minutes. After mixing by inversion, the mixture was then incubated at 70°C for 30 min. This was followed by the addition of 500 µL of chloroform-isoamylalcohol (24∶1) solution and gentle mixing by inversion for 30 seconds. Samples were then centrifuged for 4 minutes at 13,200 rpm and 500 µL of the supernatant was transferred to a fresh 2.0 mL tube. Following this 500 µL of the chloroform-isoamylalcohol (24∶1) and 200 µL 2% CTAB buffer were added to the supernatant and the solution was mixed gently by inversion. The mixture was then centrifuged for 4 minutes at 13,200 rpm and the supernatant was transferred to a fresh 1.5 mL tube with 700 µL of cold isopropanol (−20°C). Samples were gently mixed by inversion and centrifuged at 13,200 rpm for 4 minutes. After centrifugation, the supernatant was discarded and the resulting pellet was air-dried for 5 minutes. The pellet was then washed with 700 µL of cold 70% ethanol (−20°C) to clean the DNA, vortexed and centrifuged at 13,200 rpm for 4 min. The ethanol was then discarded and the pellet was air-dried for 5 minutes. The DNA was then re-suspended in 150 µL TE 10∶1 buffer (10 mM Tris-HCl pH 8.0, 1 mM EDTA pH 8.0) plus 4 µL of ribonuclease (RNAse 10 mg/mL) and was incubated at 65°C for 8 minutes. DNA was then quantified using a Nanodrop ND-1000 spectrophotometer (Thermo Scientific Inc., Wilmington, DE, USA) prior to storage at −20°C. Samples were then diluted to 10 ng/µL with deionized distilled water for SSR amplifications and stored at −20°C.

### SSR Markers and PCR Conditions

A total of 23 SSR markers (Table 1) were selected for this study taking into consideration their Polymorphic Information Content (PIC) value which ranged from 0.14 to 0.96 and their use in previous related studies (Buteler *et al.* [12], Benavides (unpublished data CIP 2002 to 2003), Solis *et al.*, (unpublished data CIP 2005 to 2006), Tumwegamire *et al.* [16]; Yada *et al.* [17], Yañez [18]. M13-tailed primer method [19] was used in this study which consisted of using 3 primers: a forward primer (with an M13 tail sequence), a reverse primer and a fluorescently labeled M13 IRDye primer. The PCR reaction was prepared in a total volume of 25 µL and the PCR reaction was as follows: 1X reaction buffer, 7 mM MgCl_2_, 4 mM KCl, 0.4 mM dNTP′s, 2 µM from each forward and reverse primers, M13 F/d 1 pmol/µL fluorescent primer (LI-COR Bioscience, Lincoln, NE, USA), 1 U Taq polymerase and 20 ng/µL DNA template. For SSR R03, 9 mM MgCl_2_ and 30 ng/µL DNA template were used. For SSR R08, 8 mM MgCl_2_ and 40 ng/µL DNA template was used. PCR conditions for amplification were 94°C for 7 minutes followed by 35 cycles of 94°C for 30 seconds, 50–63°C for 90 seconds of annealing temperature depending on the SSR marker (Table 1), 72°C for 1 minute, with a final extension time of 10 minutes at 72°C. The PCR products were denatured and visualized on a 6.5% denaturing polyacrylamide gel in a LI-COR 4300 automated DNA analyzer (LI-COR) and each allele was scored by comparison to a 50–350 base pair size standard IRDye 700 molecular size ladder (LI-COR) which was run multiple times in each gel. The alleles were called based on visual inspection of gels considering the presence of stutter bands for some SSRs.

**Table 1:** Twenty-three SSR marker primers with their respective sequence, annealing temperatures, repeat motifs and allele size.

Name	Forward Primer	Reverse Primer	Annealing °C	Motif	Allele Size	H_O_	H_T_
IB242^a^	gcggaacggacgagaaaa	atggcagagtgaaaatggaaca	58	(ct)3ca(ct)11	136–155	0.89	0.84
IB297^a^	gcaatttcacacacaaacacg	cccttcttccaccactttca	58	(ct)13	150–182	0.76	0.84
IB324^b^	tttggcatgggcctgtatt	gttcttctgcactgcctgattc	56	-	136–152	0.73	0.78
IBCIP-1^c^	cccacccttcattccattact	gaacaacaacaaaaggtagagcag	63	(acc)7a	155–167	0.79	0.74
IBS10^d^	ctacgatctctcggtgacg	cagcttctccactccctac	60	(ct)12	307–337	0.49	0.86
IBS11^d^	ccctgcgaaatcgaaatct	ggacttcctctgccttgttg	58	(ttc)10	254–305	0.95	0.88
IBR13^d^	gtaccgagccagacaggatg	cctttgggattggaacacac	58	(ttc)6	225–298	0.73	0.88
IBS17^d^	cagaagagtacgttgctcag	gcacagttctccatcctt	58	(gga)4	182–204	0.96	0.87
IBS18^d^	ctgaacccgacagcacaag	gggaagtgaccggacaaga	58	(tagc)4	296–298	0.45	0.70
IBR21^d^	gacagtctccttctcccata	ctgaagctcgtcgtcaac	58	(gac)5	181–207	0.80	0.63
IBC12^e^	tctgagcttctcaaacatgaaa	tgagaattcctggcaaccat	56	(ttc)6	110–134	0.86	0.87
J175^e^	atctatgaaatccatcactctcg	actcaattgtaagccaaccctc	58	(aatc)4	133–149	0.87	0.88
J10a^e^	tcaaccactttcattcactcc	gtaattccaccttgcgaagc	58	(aag)6	191–225	0.79	0.85
J67^e^	cacccatttgatcatctcaacc	ggctctgagcttccattgttag	58	(gaa)5	191–217	0.94	0.80
J116a^e^	tcttttgcatcaaagaaatcca	cctcagcttctgggaaacag	58	(cct)6	207–251	0.65	0.88
IB1809^e^	cttctcttgctcgcctgttc	gatagtcggaggcatctcca	60	(cct)6(ccg)6	144–155	0.78	0.81
IBJ544b^e^	agcagttgaggaaagcaagg	caggatttacagccccagaa	61–62	(tct)5	191–214	0.76	0.66
IBS01^f^	tcctccaccagctctgattc	ccattgcagagccatacttg	56	(aga)10	233–268	0.37	0.67
IB-R16^d^	gacttccttggtgtagttgc	agggttaagcgggagact	60	(gata)4	201–203	0.63	0.61
IB-R19^f^	ggctagtggagaaggtcaa	agaagtagaactccgtcacc	60	(cag)5	190–208	0.15	0.65
IB-R03^d^	gtagagttgaagagcgagca	ccatagacccattgatgaag	58	(gcg)5	243–258	0.42	0.65
IB-R08^f^	ggcgacaccttagagtat	cacccccctattcacaa	50	(t3a)4	204–216	0.86	0.70
IB-S07^d^	gcttgcttgtggttcgat	caagtgaagtgatggcgttt	60	(tgtc)7	193–211	0.59	0.79

H_O_: Observed heterozygosity.

H_T_: Total heterozygosity.

SSR Source: ^a^ Buteler *et al.* [12]; ^b^ Tseng *et al.* [15]; ^c^ Yañez [18]; ^d^ Benavides (unpublished data; 2002–2003 at CIP); ^e^ Solis *et al.* (unpublished data; 2005–2006 developed at CIP); ^f^ Yada *et al.* [17].

### Statistical Analysis

The scored data for the 23 SSR markers were converted into a weight matrix in Microsoft Excel and further converted into text using TextPad ver. 6.2.2 (Helios Software Solutions, Longridge, England). The GenoDive program was used to determine the genetic diversity estimators [i.e. observed and total heterozygosity (H_O_ and H_T_, respectively), and inbreeding coefficient (G_is_)] and allele frequencies. Since in a hexaploid is not possible to determine the actual genotype of an individual based on molecular markers, GenoDive program uses a maximum likelihood method to correct for the unknown dosage of alleles. Briefly, for every incomplete genotype information of an individual, all possible underlying genotypes are considered, new alleles frequencies for this particular locus are determined, and the genotype that end in producing the most likelihood allele frequencies is selected. Thus, this algorithm was employed for every SSR locus in our study. From the weight data, a binary matrix was constructed based on presence and absence of alleles (1, 0) [20]. The matrix was used to determine the PIC value of SSRs and Euclidean genetic distances among accessions as implemented in PowerMarker V3.25 program [21]. The Unweighted Pair Group Method with Arithmetic Mean (UPGMA) cluster method was employed with the Euclidean genetic distances as implemented in PowerMarker V3.25 program. The dendrogram was visualized using the international Tree of Life (iTOL) program [22], [23]. The population structure group was estimated using the Bayesian cluster analysis as implemented in the Structure 2.3.3 software [24]. The admixtures model with correlated allele frequencies was implemented, with 50,000 burn-in period length and 150,000 MCMC interactions including 3 independent runs for each k set from 1 to 12. Subsequently, the number of groups was determined based on deltaK (ΔK) as implemented in Structure Harvester software [25], [26]. Lastly, independent runs from the actual number of groups were integrated by CLUMPP version 1.1.2 [27]. The analysis of molecular variance (AMOVA), employing the number of groups previously identified and the binary matrix, was performed using GeneAlEx 6.5 [28].

## Results and Discussion

**Figure 1:**
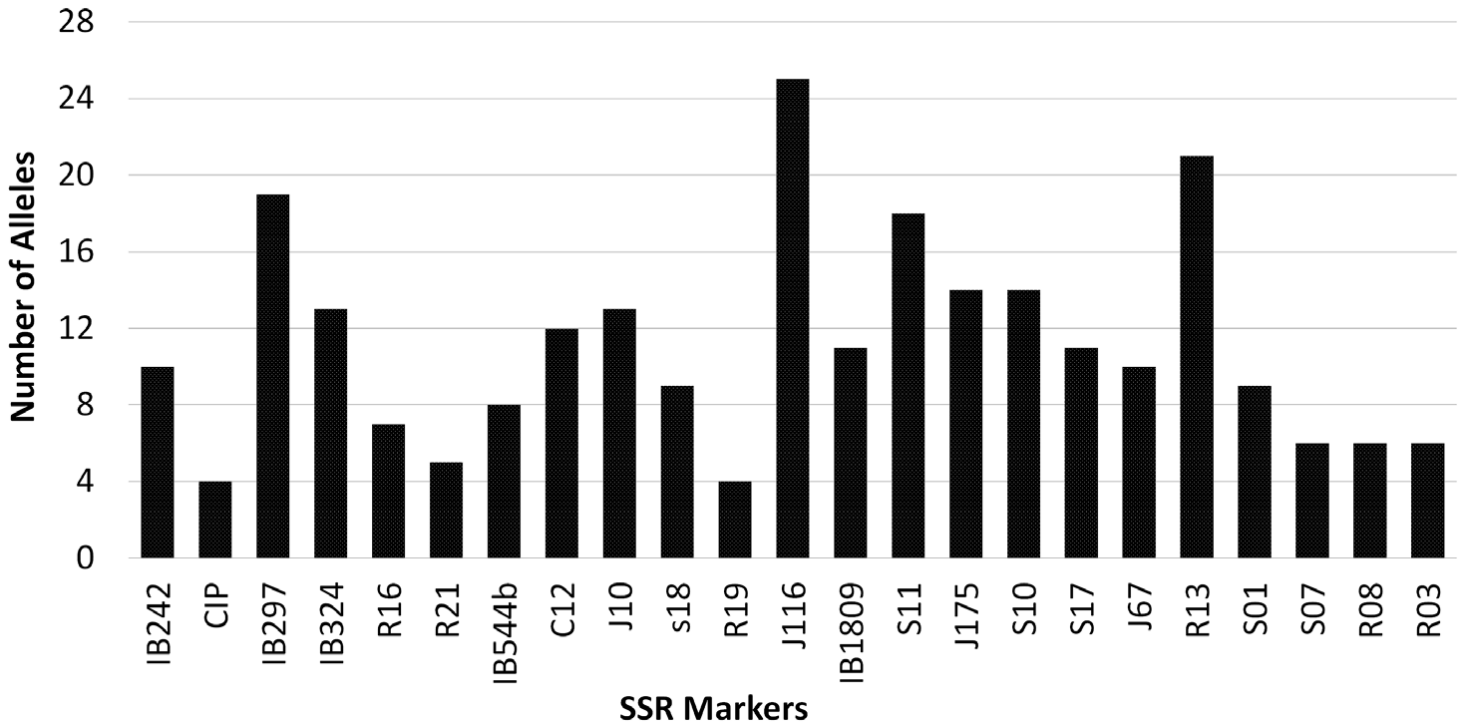
Distribution of the number of alleles per locus analyzed for 23 SSR loci in 167 accessions of sweet potato (*Ipomoea batatas*).

**Figure 2:**
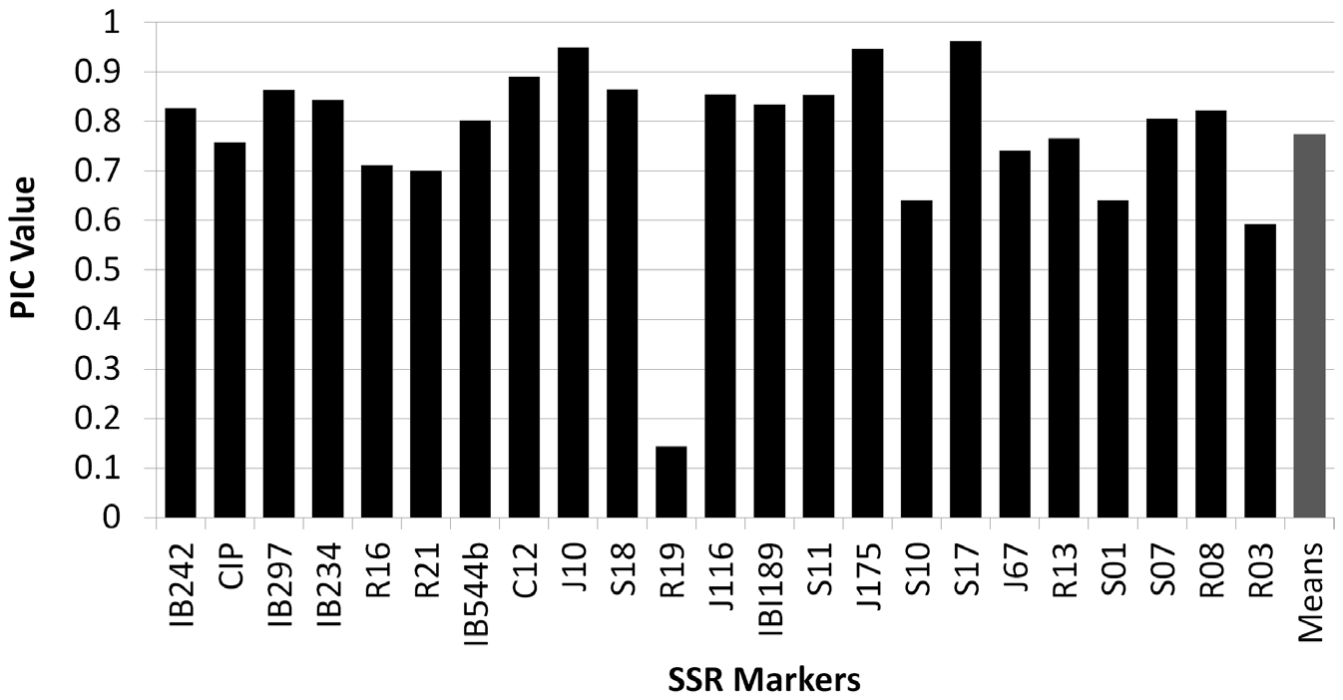
Polymorphic Index Content (PIC) value of the 23 SSR loci analyzed in 167 accessions of sweet potato (*Ipomoea batatas*).

The SSR marker analysis of the 23 loci showed a total of 255 alleles, ranging from 4 to 25 alleles per locus with a mean value of 11.08 alleles per locus (Fig. 1, Table 2). These results are comparable to others [12], [29], [30] while differing slightly with Tumwegamire *et al.* [16] who analyzed 92 African accessions with 26 SSR markers and found a mean value of 6.1 alleles per locus ranging from 2 to 11. Similarly, Gwandu *et al.* [31] analyzed 57 sweet potato genotypes in Tanzania with 4 SSR′s and found high quantities of alleles, fluctuating from 11 to 22. The high number of alleles found in sweet potato can be explained by the hexaploidy nature of sweet potato. Considering that the mean value of alleles per locus is 11.08 we can conclude with certainty that the sweet potato population in Puerto Rico is genetically very diverse. The Puerto Rican accessions in germplasm collections (GAES and PGRCU) exhibited 83 unique alleles whereas the PR landraces had 34. The US commercial cultivars showed only 6 unique alleles, sharing most of the alleles with the PR landraces. The average PIC value was 0.79 with SSRs C12 (0.89), J10 (0.95), J175 (0.95) and S17 (0.96) having the highest values (Fig. 2). The loci with the lowest PIC values were R19 (0.14) and R03 (0.59).

**Table 2:** Summary statistics of genetic diversity estimators at 23 SSR loci for 167 sweet potato samples.

Group	NoA	H_o_	H_T_	G_is_
**PR accessions (GAES and PGRCU)**	8.65	0.80	0.80	0.00
**US cultivars**	4.68	0.70	0.72	0.01
**PR Landraces**	7.18	0.66	0.72	0.08
***Groups based on STRUCTURE***				
**G1- PR-PGRCU**	6.50	0.85	0.75	−0.13
**G2- PR-GAES**	4.95	0.70	0.67	−0.03
**G3- US cultivars**	4.04	0.67	0.70	0.03
**G4**	5.63	0.69	0.71	0.03
**G5**	5.09	0.71	0.70	−0.01
**G6**	4.76	0.59	0.66	0.10
**G7**	4.49	0.68	0.67	0.00
**G8**	4.72	0.54	0.71	0.23
**Mean**	11.08	0.71	0.78	0.05
**Std. Dev.**	1.15	0.04	0.02	0.04

(NoA: Number of Alleles, H_o_: Observed Heterozygosity, H_T_: Total Heterozygosity, G_is_: Inbreeding Coefficient).

The H_O_ was 0.71, ranging from 0.15 to 0.95 between loci (Table 1, Table 2). Previous studies in sweet potato also found high levels of heterozygosity with values of 0.37 for Tropical America, 0.60 for Latin American and 0.75 for Kenyan accessions [9], [30], [32]. In sweet potato high levels of heterozygosity and genetic diversity could be explained by the outcrossing and self-incompatible nature of the plant. For instance, this self-incompatibility in the field might result in chance seedlings from crossings, providing another path to increase genetic diversity [17]. The group comprised of the PR accessions from GAES had the highest value of H_O_ (0.81) followed by the PR accessions from PGRCU (0.71) and the PR landraces (0.66) (Table 2). These high levels of heterozygosity found in germplasm collection and landraces could be further explained by the possibility that this sweet potato is a descendant from one of the original domestication events that occurred in the northern part of South America such as Venezuela and brought to the West Indies by the Arawak Indians who populated the area. If indeed sweet potato was introduced to Puerto Rico more than 500 years ago, additional ecological factors such as climate and soil conditions as well as human practices might have added more diversity to this already complex and self-incompatible crop. The GAES collection, which exhibited high values of observed heterozygosity, is mainly composed of sweet potato varieties of economic importance in the US. These accessions are the product of human selection which might have enhanced their genetic makeup.

**Figure 3:**
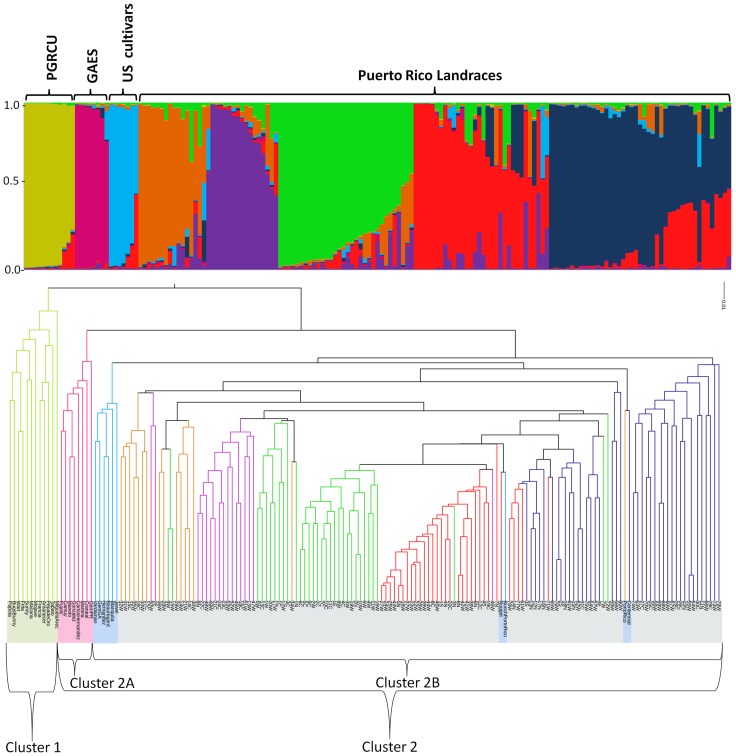
Population structure and UPGMA clustering based on Euclidean distances of 167 accessions of sweet potato (*Ipomoea batatas*) analyzed with 23 SSR markers. Eight accessions are from the agricultural experimental station in Gurabo, Puerto Rico (GAES), 22 from the plant genetic resources conservation unit (PGRCU) in Griffin, GA (12 PR accessions and 10 known US commercial cultivars) and 137 Puerto Rico landraces across the island were analyzed. The eight groups (Group 1: olive green, Group 2: pink, Group: navy blue, Group: orange, Group: purple, Group: lime green, Group: red, and Group: blue) were determined based on STRUCTURE and Evanno *et al*. [26] analysis. The colors of the branches in the dendrogram also indicates the groups while the highlight of the name refers to PRGCU (olive green), GAES (pink), US cultivars (navy blue) and PR landraces (light gray).

**Figure 4:**
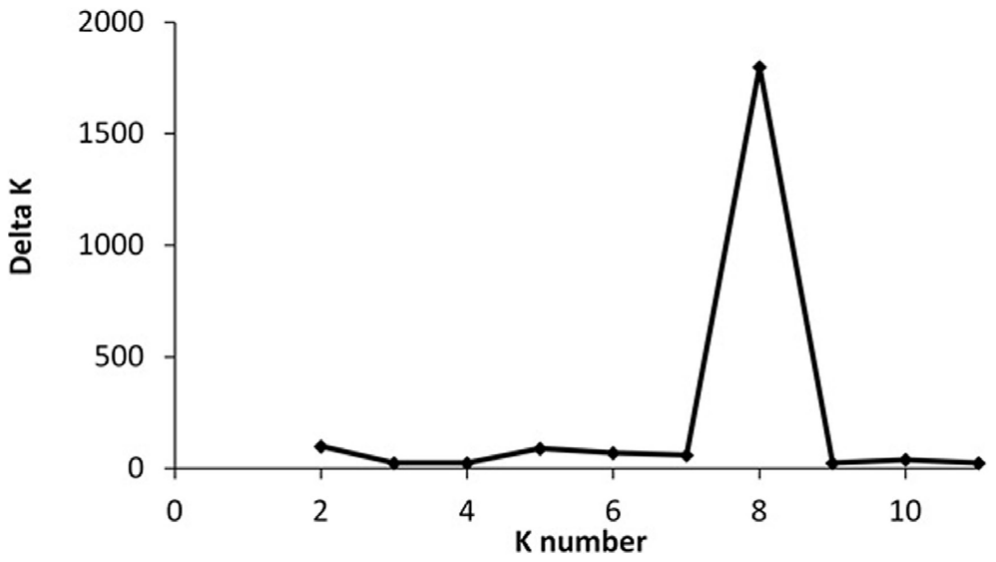
Delta K values with respect to K (number of groups) according to the calculation method by Evanno *et al*. [26]. These results were obtained based on the analysis of 23 SSR markers in 167 accessions of sweet potato from the agricultural experimental station in Gurabo, Puerto Rico (12 accessions), the plant genetic resources conservation unit in Griffin, GA (22 accessions) and 137 Puerto Rico landraces.

The result of the UPGMA clustering method using Euclidean distances as seen on the tree depicts two main clusters (Fig. 3). Cluster 1 is composed of 12 PR accessions that were collected in PR between 1986 and 1989 and is being maintained *in-vitro* in the USDA PGRCU. Cluster 2, with an overall mean value of 76% similarity, could be separated into cluster 2A and 2B. The cluster 2A is composed of the PR collection at GAES, while cluster 2B was a separation of the majority of the US cultivars from GAES and PR landraces. This result was in agreement with the population structure analysis which estimated the number of groups as eight based on ΔK (Fig. 4). Indeed, cluster 1 and 2A correspond to groups 1 and 2, respectively, while cluster 2B was divide in to six groups (group 3–8) including one group of US cultivars from GAES (group 3) and five groups enclosing PR landraces and the US cultivars Bunch Porto Rico, Porto Rico, Nugget and Centennial (group 4–8). The AMOVA determined that 25% and 75% of the variation is among and within these 8 groups, respectively. Measurements of H_T_ revealed a large genetic diversity with a mean value of 0.78 and a range from 0.62 to 0.88 per locus (Table 1). The H_T_ within groups ranged from 0.66 to 0.80 revealing high levels of genetic diversity (Table 2). Our results are comparable with those of Gwandu *et al.* [31] and Tumwegamire *et al.* [16] who found that the main source of variation is within populations and those of Zhang *et al.* [9] who calculated an among populations variation of 10% and a within population variation of 90%. The resulting inbreeding coefficient (G_is_) was low with a value of 0.05, however, it suggests some level of inbreeding across the groups (Table 2).

The accessions of cluster 1 (group 1) were strategically selected to represent the genetic diversity present in PR and has been maintained *in vitro* by clonal propagation, maintaining the individual genetic composition and variability, but also avoiding the incorporation of new alleles. This group had the highest genetic diversity reflected by the high H_O_ and H_T_ values (0.85 and 0.75, respectively). In addition, it has the highest percentage of unique alleles (20%) among the 8 groups reflecting the genetic diversity of the collection and its adequate preservation. In this group a trend could be inferred with most of the accessions that grouped more closely share morphological traits such as root skin, flesh color and their genetic distance grouping them closely together with a mean value of 73% similarity. ‘Miguela-Arecibo’ was the first accession to be introduced to the PGRCU germplasm in 1986, interestingly it is the only sample with purple flesh (root skin color is red) in cluster 1 and grouped separately from the rest. Others such as Tapato, Amanecer, Pepa de Oro, Frita, Wart and Francia have yellow/golden root skin color. Accessions with red/orange fleshed roots such as Macana and Sunny also grouped together. Bugs Bunny and Papota have cream colored root flesh, while Mojave is the only white skin/flesh accession.

The PR collection at GAES (cluster 2A: group 2) is being maintained in the field through vegetative propagation and gene flow among them is constantly occurring by cross pollination. This group had 10% of unique alleles, and high levels of genetic diversity reflected by the high H_O_ and H_T_ values (0.70 and 0.67, respectively). The lower percentage of unique alleles and genetic diversity in comparison to group 1 could be the result of genetic drift and inbreeding since this collection is maintained in open pollinated field. Another explanation could be that the collection localities of the original accessions are in close proximity to each other. Even though accessions from PR collection at GAES are also closely related to the PR landraces, they grouped separately.

The US cultivars from GAES (group 3) are widely consumed and grown by small scale farmers. The genetic variation of this group is lower than group 1 and 2 with only 2% of unique alleles, and H_O_ and H_T_ values of 0.67 and 0.70, respectively. Likewise, the percentages of unique alleles in group 4 to 8, which comprise PR landraces and four US cultivars, were lower than the observed in PGRCU and GAES collection. The H_O_ values across these five groups ranged from 0.54 to 0.71, while the total heterozygosity ranged from 0.66 to 0.71. The PR landraces are outcrossing in the field and maintained by natural selection. Thus, the low percentages of rare alleles and the observed population structure might be the result of hundreds of years of natural selection. Remarkably, PR landraces did not exhibit any kind of grouping pattern according to their geographical location. This phenomenon has been observed previously in almost all genetic diversity analysis of sweet potato [16], [17], [31], [32], [33]. Yada *et al.* [17] reported results similar in which samples clustered randomly and did not follow any distinguishable order, suggesting that a significant exchange of material is happening between landowners and farmers. Clustering of samples not due to geographical locations could be due to the ease of sweet potato propagation which is performed using either the stem parts or the root. Another cause could be the self-incompatibility of sweet potato which prevents inbreeding and allele fixation, facilitating new natural combinations to occur. According to Bruckner [34], accessions can group together but display very little if any regional similarity, suggesting the presence of an extremely high amount of genetic diversity in the sweet potato gene pool. Although these groups could not be associated with their geographical origins, further research should be directed to determine the factors defining this population structure.

Most of the commercial varieties from the US grouped closely with the PR landraces (Fig. 3). Some of these accessions are widely used and consumed in the US and there is a possibility that the accessions such as Nugget, Centennial, Bunch PR and Porto Rico are being grown all over the island for personal consumption. In fact, these cultivars are some of the more desirable cultivars for consumption due to their flavor, texture, sweetness and consistency. Interestingly, all of them are orange fleshed cultivars. They could have been introduced to the island and cultivated by homeowners or small scale farmers, thus intercrossing with the PR landraces. Also some of these accessions have Puerto Rican genetic background as one of the parents used in the breeding process was from the PR collection at PGRCU, resulting in the sharing of alleles between PR landraces and these US cultivars.

In conclusion, from our study it can be observed that the ability of dispersal of a vegetatively propagated crop can lead to genetic diversity preservation and increase. Farmers and landowners are fundamental in the diffusion of such crops. Unconsciously, the farmers maintain healthy levels of plant genetic diversity, when introducing different accessions into the field (by material exchange or importation), and later by gene flow, recombination and somatic variation the genetic diversity of the crop increases and is preserved by natural and agronomic selection. High levels of genetic diversity found in sweet potato in Puerto Rico, the history of domestication and the methods of dispersal of sweet potato suggest that Puerto Rico may be one of the secondary centers for diversity of sweet potato. This adds to the importance of protecting and further studying these genetic resources in the Caribbean.

## Supporting Information

S1 Table **Information of the sweet potato samples used to study the genetic diversity present in Puerto Rico.** Name (ID), source, collection site, and status of sweet potato materials used are shown. Eight accessions are from the agricultural experimental station in Gurabo, Puerto Rico (GAES), 22 from the USDA plant genetic resources conservation unit (PGRCU) in Griffin, GA (12 PR accessions and 10 known US commercial cultivars) and 137 Puerto Rico landraces are from across the island.
(DOCX)
