# Supplementary material for: Assessment of Genetic Diversity of Sweet Potato in Puerto Rico
Source: PLoS One. 2014 Dec 31;9(12):e116184. doi: 10.1371/journal.pone.0116184 (PMC4281141; doi:10.1371/journal.pone.0116184)
Supplement: S1 Table — Information of the sweet potato samples used to study the genetic diversity present in Puerto Rico. Name (ID), source, collection site, and status of sweet potato materials used are shown. Eight accessions are from the agricultural experimental station in Gurabo, Puerto Rico (GAES), 22 from the USDA plant genetic resources conservation unit (PGRCU) in Griffin, GA (12 PR accessions and 10 known US commercial cultivars) and 137 Puerto Rico landraces are from across the island. (DOCX) [file pone.0116184.s001.docx]

**Table S1**. Information of the sweet potato samples used to study the genetic diversity present in Puerto Rico. Name (ID), source, collection site, and status of sweet potato materials used are shown. Eight accessions are from the agricultural experimental station in Gurabo, Puerto Rico (GAES), 22 from the USDA plant genetic resources conservation unit (PGRCU) in Griffin, GA (12 PR accessions and 10 known US commercial cultivars) and 137 Puerto Rico landraces are from across the island.

| **ID** | **Source** | **Collection Site** | **Status** |
| --- | --- | --- | --- |
| Gonzalez | GAES | Collected, Puerto Rico | PR Accession |
| Martina | GAES | Collected, Puerto Rico | PR Accession |
| Camuy | GAES | Collected, Puerto Rico | PR Accession |
| Manolo | GAES | Collected, Puerto Rico | PR Accession |
| Gem | GAES | Collected, Puerto Rico | PR Accession |
| Craneal | GAES | Collected, Puerto Rico | PR Accession |
| Carlos Hernandez | GAES | Collected, Puerto Rico | PR Accession |
| Pujols | GAES | Collected, Puerto Rico | PR Accession |
| GemGA | PGRCU | Collected, United States | US Cultivar |
| Jewel | PGRCU | Developed. North Carolina, United States. | US Cultivar |
| Blanquita | PGRCU | Donated, Puerto Rico | US Cultivar |
| Beauregard | PGRCU | Developed. Louisiana, United States | US Cultivar |
| Hernandez | PGRCU | Developed. Louisiana, United States | US Cultivar |
| Vardaman | PGRCU | Developed. Mississippi, United States | US Cultivar |
| Centennial | PGRCU | Developed. Louisiana, United States | US Cultivar |
| Porto Rico | PGRCU | Developed, North Carolina, United States | US Cultivar |
| Nugget | PGRCU | Developed. North Carolina, United States | US Cultivar |
| Bunch Porto Rico | PGRCU | Donated, Louisiana, United States | US Cultivar |
| Miguela-Arecibo | PGRCU | Collected, Puerto Rico | PR Accession |
| Tapato | PGRCU | Collected, Puerto Rico | PR Accession |
| Pepa de Oro | PGRCU | Collected, Puerto Rico | PR Accession |
| Amanecer | PGRCU | Collected, Puerto Rico | PR Accession |
| Francia | PGRCU | Collected, Puerto Rico | PR Accession |
| Mojave | PGRCU | Collected, Puerto Rico | PR Accession |
| Macana | PGRCU | Collected, Puerto Rico | PR Accession |
| Sunny | PGRCU | Collected, Puerto Rico | PR Accession |
| Frita | PGRCU | Collected, Puerto Rico | PR Accession |
| Wart | PGRCU | Collected, Puerto Rico | PR Accession |
| Buggsbunny | PGRCU | Collected, Puerto Rico | PR Accession |
| Papota | PGRCU | Collected, Puerto Rico | PR Accession |
| 1W | Land owners | Añasco | PR Landraces |
| 2W | Land owners | Mayaguez | PR Landraces |
| 3W | Land owners | Isabela | PR Landraces |
| 1N | Land owners | Guaynabo | PR Landraces |
| 2N | Land owners | Camuy | PR Landraces |
| 3N | Land owners | Vega Alta | PR Landraces |
| 1C | Land owners | Lares | PR Landraces |
| 4W | Land owners | Lajas | PR Landraces |
| 2C | Land owners | Jayuya | PR Landraces |
| 5W | Land owners | Añasco | PR Landraces |
| 3C | Land owners | Caguas | PR Landraces |
| 6W | Land owners | Aguadilla | PR Landraces |
| 7W | Land owners | Aguadilla | PR Landraces |
| 8W | Land owners | Moca | PR Landraces |
| 4C | Land owners | Cidra | PR Landraces |
| 9W | Land owners | Aguada | PR Landraces |
| 1E | Land owners | Gurabo | PR Landraces |
| 10W | Land owners | Aguadilla | PR Landraces |
| 11W | Land owners | Cabo Rojo | PR Landraces |
| 12W | Land owners | Aguada | PR Landraces |
| 13W | Land owners | Aguada | PR Landraces |
| 14W | Land owners | Aguada | PR Landraces |
| 15W | Land owners | Aguada | PR Landraces |
| 16W | Land owners | Sabana Grande | PR Landraces |
| 17W | Land owners | Isabela | PR Landraces |
| 4N | Land owners | Barceloneta | PR Landraces |
| 18W | Land owners | Moca | PR Landraces |
| 19W | Land owners | San German | PR Landraces |
| 5C | Land owners | Utuado | PR Landraces |
| 6C | Land owners | Morovis | PR Landraces |
| 7C | Land owners | Morovis | PR Landraces |
| 8C | Land owners | Morovis | PR Landraces |
| 9C | Land owners | Morovis | PR Landraces |
| 10C | Land owners | Morovis | PR Landraces |
| 11C | Land owners | Morovis | PR Landraces |
| 12C | Land owners | Morovis | PR Landraces |
| 13C | Land owners | Morovis | PR Landraces |
| 20W | Land owners | Sabana Grande | PR Landraces |
| 21W | Land owners | Añasco | PR Landraces |
| 14C | Land owners | Lares | PR Landraces |
| 22W | Land owners | Mayaguez | PR Landraces |
| 23W | Land owners | Mayaguez | PR Landraces |
| 24W | Land owners | Mayaguez | PR Landraces |
| 5N | Land owners | Manati | PR Landraces |
| 25W | Land owners | Isabela | PR Landraces |
| 26W | Land owners | Rincon | PR Landraces |
| 27W | Land owners | Mayaguez | PR Landraces |
| 6N | Land owners | Bayamon | PR Landraces |
| 28W | Land owners | Hormigueros | PR Landraces |
| 29W | Land owners | Lajas | PR Landraces |
| 30W | Land owners | San German | PR Landraces |
| 15C | Land owners | Lares | PR Landraces |
| 16C | Land owners | Lares | PR Landraces |
| 17C | Land owners | Lares | PR Landraces |
| 31W | Land owners | Lajas | PR Landraces |
| 32W | Land owners | Lajas | PR Landraces |
| 33W | Land owners | Quebradillas | PR Landraces |
| 34W | Land owners | Quebradillas | PR Landraces |
| 35W | Land owners | Quebradillas | PR Landraces |
| 36W | Land owners | Aguada | PR Landraces |
| 37W | Land owners | Cabo Rojo | PR Landraces |
| 38W | Land owners | Mayaguez | PR Landraces |
| 2E | Land owners | Gurabo | PR Landraces |
| 18C | Land owners | Ciales | PR Landraces |
| 19C | Land owners | Corozal | PR Landraces |
| 39W | Land owners | Mayaguez | PR Landraces |
| 40W | Land owners | Moca | PR Landraces |
| 7N | Land owners | San Juan | PR Landraces |
| 20C | Land owners | Aibonito | PR Landraces |
| 41W | Land owners | Mayaguez | PR Landraces |
| 8N | Land owners | Camuy | PR Landraces |
| 42W | Land owners | Guanica | PR Landraces |
| 43W | Land owners | Moca | PR Landraces |
| 44W | Land owners | Sabana Grande | PR Landraces |
| 45W | Land owners | Mayaguez | PR Landraces |
| 46W | Land owners | San Sebastian | PR Landraces |
| 9N | Land owners | Hatillo | PR Landraces |
| 21C | Land owners | Corozal | PR Landraces |
| 47W | Land owners | San German | PR Landraces |
| 48W | Land owners | San Sebastian | PR Landraces |
| 22C | Land owners | Barranquitas | PR Landraces |
| 49W | Land owners | Moca | PR Landraces |
| 50W | Land owners | Añasco | PR Landraces |
| 51W | Land owners | Aguada | PR Landraces |
| 52W | Land owners | Cabo Rojo | PR Landraces |
| 23C | Land owners | Barranquitas | PR Landraces |
| 53W | Land owners | Aguada | PR Landraces |
| 24C | Land owners | Corozal | PR Landraces |
| 54W | Land owners | Isabela | PR Landraces |
| 55W | Land owners | Isabela | PR Landraces |
| 25C | Land owners | Utuado | PR Landraces |
| 56W | Land owners | Aguada | PR Landraces |
| 26C | Land owners | Comerio | PR Landraces |
| 10N | Land owners | Carolina | PR Landraces |
| 3E | Land owners | Rio Grande | PR Landraces |
| 57W | Land owners | Aguadilla | PR Landraces |
| 11N | Land owners | Camuy | PR Landraces |
| 58W | Land owners | Isabela | PR Landraces |
| 1S | Land owners | Patillas | PR Landraces |
| 59W | Land owners | San Sebastian | PR Landraces |
| 60W | Land owners | Mayaguez | PR Landraces |
| 61W | Land owners | Isabela | PR Landraces |
| 62W | Land owners | San Sebastian | PR Landraces |
| 63W | Land owners | Moca | PR Landraces |
| 64W | Land owners | Mayaguez | PR Landraces |
| 65W | Land owners | Mayaguez | PR Landraces |
| 27C | Land owners | Jayuya | PR Landraces |
| 66W | Land owners | Isabela | PR Landraces |
| 67W | Land owners | Aguada | PR Landraces |
| 68W | Land owners | Aguada | PR Landraces |
| 69W | Land owners | Cabo Rojo | PR Landraces |
| 28C | Land owners | Utuado | PR Landraces |
| 70W | Land owners | Cabo Rojo | PR Landraces |
| 29C | Land owners | Caguas | PR Landraces |
| 71W | Land owners | Sabana Grande | PR Landraces |
| 72W | Land owners | San German | PR Landraces |
| 73W | Land owners | Isabela | PR Landraces |
| 74W | Land owners | Moca | PR Landraces |
| 12N | Land owners | Bayamon | PR Landraces |
| 13N | Land owners | Bayamon | PR Landraces |
| 75W | Land owners | San German | PR Landraces |
| 76W | Land owners | Añasco | PR Landraces |
| 2S | Land owners | Yauco | PR Landraces |
| 14N | Land owners | Manati | PR Landraces |
| 15N | Land owners | Camuy | PR Landraces |
| 77W | Land owners | Moca | PR Landraces |
| 78W | Land owners | Guanica | PR Landraces |
| 79W | Land owners | Aguadilla | PR Landraces |
| 80W | Land owners | San Sebastian | PR Landraces |
| 16N | Land owners | Vega Baja | PR Landraces |
| 30C | Land owners | Lares | PR Landraces |
| 3S | Land owners | Yauco | PR Landraces |
| 4S | Land owners | Coamo | PR Landraces |
| 31C | Land owners | Barranquitas | PR Landraces |
| 17N | Land owners | Camuy | PR Landraces |
| 81W | Land owners | Las Marias | PR Landraces |
| 18N | Land owners | San Juan | PR Landraces |
